# Early Technology Readiness Level (TRL) Development of the Microfluidic Inorganic Conductivity Detector for Europa and the Solenoid-Based Actuator Assembly for Impact Penetrators

**DOI:** 10.3390/s24237704

**Published:** 2024-12-02

**Authors:** Chinmayee Govinda Raj, Mohamed Odeh, Cambrie Salyards, Amanda Stockton

**Affiliations:** Georgia Institute of Technology, School of Chemistry and Biochemistry, 901 Atlantic Dr. NW, Atlanta, GA 30332, USA

**Keywords:** Europa, instrumentation, exobiology, solar system origin, prebiotic chemistry

## Abstract

This study introduces an innovative in situ lander/impact-penetrator design tailored for Discovery-class missions to Europa, specifically focused on conducting astrobiological analyses. The platform integrates a microfluidic capacitively coupled contactless conductivity detector (C4D), optimized for the detection of low-concentration salts potentially indicative of biological activity. Our microfluidic system allows for automated sample routing and precise conductivity-based detection, making it suitable for the harsh environmental and logistical demands of Europa’s icy surface. This technology provides a robust toolset for exploring extraterrestrial habitability by enabling in situ chemical analyses with minimal operational intervention, paving the way for advanced astrobiological investigations on Europa.

## 1. Introduction

Europa emerged as a top-priority astrobiological target following the discovery of its global subsurface saline ocean and an enhanced understanding of its geochemistry and thermodynamics through remote sensing [1,2,3]. Remote sensing techniques are powerful tools for extraterrestrial exploration, but in situ data through analyses of subsurface materials are often necessary for habitability investigations [4]. Amino acids have been found in low abundances in multiple extraterrestrial samples [5,6]; their chirality and compositional information can serve as potential biomarkers [7,8]. The ability to identify these abundances can help inform Europa’s prebiotic chemistry, habitability, and history [9]. Similarly, understanding the origin of salts on the surface of Europa has pivotal implications for understanding the habitability of its ocean [10,11]. Unfortunately, determining the provenance of ionic species has proven particularly difficult due to incongruent data. MgCl_2_, NaCl, and KCl are the dominating salts based on data from Keck [10], while data from Galileo suggest MgSO_4_ and Na_2_SO_4_. Adding to this contrast, sulfates on Europa’s surface are hypothesized to potentially be byproducts of Io’s volcanic activity [12,13], while computational models suggest otherwise [14,15]. This ambiguity can be addressed only by gaining information on exogenic ion impact depth over a wide geographical area distribution.

The Europa Lander mission concept design could be the first mission to “ground truth” and look for signs of life in the icy crust of Europa [4]. Lander missions are typically Flagship missions and therefore carry heavy, high-volume, power-hungry, and fragile instrument suites requiring detailed, multi-step soft-landing architectures. This adds to the complexity and demands extensive time and resource allotment for development, launch, and operation. The Europa Lander would further require specialized ice-drilling equipment for the extraction of radiation-shielded samples beneath the surface [4,9] but would be stationary, without providing geochemical spatial distribution information. The Voyager and Galileo missions have helped recognize that Europa has highly disrupted areas, dubbed as “chaotic terrain”, ranging over a size range of at least three orders of magnitude, from km scale features to 1300 km across [16]. Hence, the functionality of a rover platform could be limited and even risk its performance due to the current lack of detailed surface maps.

As an alternative, penetrator mission architectures could be employed for in situ exploration. Penetrators are compact payloads that kinetically penetrate the crust to some depth by surviving high g impact loads and conduct analyses on board. Penetrator designs with electronic hardware have excellent heritage with military systems, and now, instrumented penetrator designs for planetary exploration are gaining interest due to their potential for low-cost, high-quality science data return [17,18,19,20]. Analytical devices that can detect and identify amino acids and salts with an instrument suite fitting within a low-volume, -mass, and -power envelope while also being robust to high g load accelerations are essential for instrumented penetrator platforms.

The advent of microfluidic technology has made rapid analyses with small sample volumes easily accomplishable with low power needs [21]. At the heart of these microfluidic devices are sample processors that often employ pneumatically actuated microvalves [22,23]. Multiple microvalves in an array can be used to form a peristaltic pump to facilitate fluid intake, routing, mixing, counting, dilution, reaction, etc., to form a programmable microfluidic platform (PMP) [22]. These PMPs can be integrated with embedded microsensing capabilities for applications like DNA detection [24], wearable biofluid sensors [25], and even a multitude of sensors on one chip [26]. Ionic detection in microfluidic platforms predominantly uses capacitively coupled contactless conductivity detectors (C^4^D), including PMPs for point-of-care medical diagnostics [27] and environmental analysis devices in the field [28,29], and these have been proposed for space mission applications [30,31].

The Ice Shell Impact Penetrator (IceShIP) is a microfluidic instrument designed to enable instrumented impact-penetrator missions. A subpayload of IceShIP is the Icy Moon Penetrator Organic Analyzer (IMPOA), which uses laser-induced fluorescence (LIF) to detect low-concentration organic species and is capable of surviving a 50 kg impact force [18]. With this capability, IMPOA can completely avoid soft-lander platforms, impact the surface, and penetrate a few meters to protected, near-surface samples. A recent addition to IceShIP is the Microfluidic Inorganic Conductivity detector for Europa (MicroICE) [32] for quantifying low-concentration inorganics on a microfluidic platform.

In this work, we demonstrate the performance of a miniaturized MicroICE prototype and test the device with seven “real-world” samples to validate the device’s performance. We integrate the MicroICE device with automated sample inlet and routing capabilities through the Solenoid-based actuator assembly for Impact Penetrators (SIP). The SIP uses commercially available off-the-shelf (COTS) components with geometries suitable for integration with penetrator-type payloads. MicroICE and SIP devices are supported by a low-power, small-footprint electronic hardware circuit built for the IceShIP canister. Simple fabrication with the usage of COTS components makes technology transfer for flight applications quick and uncomplicated. We demonstrate the setup at TRL 3 with a low-mass, low-power, small-volume instrument design geared towards high-acceleration space flight mission design. The IMPOA and MicroICE equipped with SIP within a single miniaturized payload design discussed in this work could help quantify organic and inorganic content and gather in situ data from multiple geographical locations for enhanced science return informing about Europa’s geochemistry.

## 2. Concept

**Figure 1:**
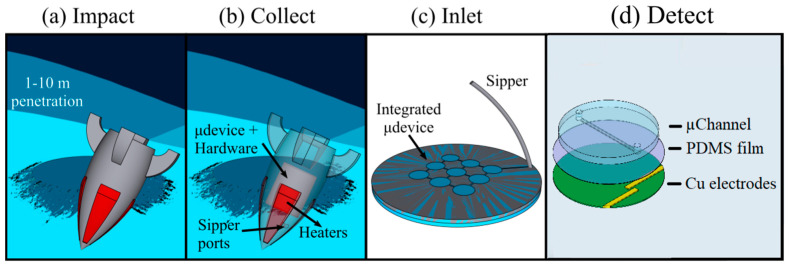
Schematic of the sequence of operations of a MicroICE payload for detection of salts following impact, penetration, and sample internalization. The sequence of operation is as follows: (**a**) Penetrator body impacting and penetrating 1–10 m into the icy surface. (**b**) Panel showing the heaters placed around the nosecone of the impactor body turning on to melt the ice immediately around the penetrator body. Sipper ports placed in proximity to the heaters sip in molten ice and place it inside the microfluidic device (µdevice) with the placement of hardware to support melting, sipping, and detection (upcoming in the next panels). (**c**) Sipped sample placed inside the integrated microfluidic device (µdevice) for (**d**) detection using the C^4^D device. Modified from Govinda Raj et al. [32] with permission.

The complete impact-penetrator mission concept is shown in Figure 1. Multiple IceShIP canisters containing miniaturized analytical instruments could ride on a single Discovery-class orbiter and be ejected at different time intervals during the orbit to impact Europa’s surface and analyze samples with significant geographical spacing without roving capabilities. The impact force would facilitate penetration of one to several meters into the ice crust, depending on the surface properties and sabot mass and geometry (Figure 1a). Heaters on the sabot body could enable melt flow (Figure 1b). An automated peristaltic pumping mechanism could intake liquid samples into the integrated microfluidic device (Figure 1c) and route the samples within the microfluidic device, facilitating sample placement above the C^4^D electrodes for detection (Figure 1d).

The Microfluidic Inorganic Conductivity detector for Europa (MicroICE) architecture involves a pair of electrodes underneath a microchannel, with an insulating layer sandwiched in between. The electrodes function as the two plates of a capacitor, and the contents of the microchannel act as the dielectric medium—the concentration of ions in the medium dictates the capacitive coupling between the electrodes [33]. The physical isolation of the electrodes from the salt sample would enable a rapid, contamination-free, highly repeatable detection technique [32,34].

## 3. Materials and Methods

### 3.1. Reagent Preparation

Lab faucet water was triply filtered to reach a resistivity of 18 MΩ/cm and was used to prepare all aqueous stock samples. All salt stock solutions were stored at 4 °C. Copper etching solution was made using 45 mL de-ionized (DI) water, 10 mL 6 N hydrochloric acid (VWR, Solon, OH, USA), and 5 mL 30% hydrogen peroxide (Sigma Aldrich, St. Louis, MO, USA). Sodium chloride, magnesium sulfate, potassium chloride, and sodium sulfate salts were used as received (VWR, Solon, OH, USA). Food coloring solutions (McCormic, Hunt Valley, MD, USA) were diluted with DI water (1:1 ratio) to visually observe the fluidic flow inside the microchannel.

About 15 mL of SYLGARD 184 (Dow Corning, Midland, MI, USA) was prepared by thoroughly mixing 10 parts elastomer base and 1 part curing agent by volume in a 50 mL Falcon conical centrifuge tube (Fisher Scientific, Waltham, MA, USA) and degassed until bubbling subsided (~40 min).

Seven “real-world” samples were tested on Device II—tap water from the lab faucet, rainwater, and snowmelt from Georgia, USA, snow from Mt. Rainier (Washington, DC, USA), river water from Rio Tinto (Huelva, Spain), sediment samples from Dyngjusandur (Iceland), and ocean water samples (San Diego, CA, USA). Tap water was simply filled from the faucet into a Falcon tube. Rainwater was collected from a puddle, filtered, and pipetted into a microcentrifuge tube. Mt. Rainier samples were scooped into Falcon tubes and stored at −20 °C; they were melted before use. Snow was collected in three dishes placed 1 m apart over a span of 2 h and then melted and pipetted into Falcon tubes, stored at 4 °C. Rio Tinto samples were collected in Falcon tubes from the river and stored at −80 °C; they were thawed before use. For Dyngjusandur, 100 µL of DI was added to 1 g of sediment, vortexed for 10 sec, and centrifuged at 1000 RPM for 1 min. The top layer of water was pipetted for use with the device. The stock sediment was stored at −80 °C. Ocean water samples were from the Pacific Ocean and collected from the beach in San Diego; they were stored at −20 °C and thawed before use. During device validation, tap water was diluted 1:1, the Rio Tinto river sample was diluted 1:1500 and 1:750, the Dyngjusandur sample was diluted 1:1, and the ocean water sample was diluted 1:1500 and 1:750. The rainwater, snowmelt, and Mt. Rainier samples were used at their original concentrations.

### 3.2. MicroICE Subpayload

#### 3.2.1. MicroICE Electrode Fabrication

MicroICE device electrode fabrication is described in extensive detail in Govinda Raj et al. [32]. Briefly, PVC tape was used as the mold for the PDMS microchannels. Copper-clad FR-4 sheets (McMaster-Carr, Elmhurst, IL, USA) were cut into 4.8 cm diameter disks using a vertical band saw (Grizzly Industrial, Bellingham, WA, USA). PVC tape was used as the mask to make the excitation and sensing electrodes on the FR-4 sheets and immersed in copper etching solution for ~40 min. Electrical contact wires were then soldered onto the copper electrodes. We could avoid the use of the typical Faraday shield between the electrodes by using antiparallel electrode placement and empirically determined electrode widths, electrode separation gaps, and excitation signal amplitude, as suggested by Kubáň et al. [35]. The electrode geometry was measured using a microscope (Dino-lite, New Taipei City, Taiwan).

#### 3.2.2. MicroICE Device Miniaturization

A breadboard version of the C^4^D hardware was built for initial tests, followed by the printed circuit board (PCB) version using a commercial PCB plotter (LPKF Laser & Electronics, Model: ProtoMat S, Fürth, Germany) and laser etching (LPKF Laser & Electronics, Model: ProtoLaser U4, Fürth, Germany). The dimensions of the board were dictated by the IceShIP canister and therefore had a footprint of 9 × 4.5 cm^2^. The breadboard and the PCB hardware performances were compared by making calibration plots for two lab-prepared salts on the same MicroICE device.

#### 3.2.3. MicroICE LOD Measurement Technique

The MicroICE device LOD measurement technique is described in extensive detail in Govinda Raj et al. [32]. In short, for all four salts of interest, three independent 1M stock solutions were prepared and serially diluted to eight samples: 0, 10 μM, 30 μM, 50 μM, 100 μM, 300 μM, 500 μM, and 1000 μM. For data post-processing, one-step calibration entailed offsetting voltages corresponding to DI water (0 μM concentration) to 0 V. The LOD was calculated by linear fitting the data, then calculating the concentration at an SNR equal to 3.

#### 3.2.4. MicroICE Hardware Setup, Operation, and Data Processing

The detector hardware settings and circuitry are discussed in extensive detail in Govinda Raj et al. [32]. Briefly, an Arduino NANO (ATtmega328, Sommerville, MA, USA) was programmed to generate a pulse-width-modulated (PWM) signal (PN: Si5351, Adafruit, New York, NY, USA) at 560 kHz. The output square wave was modified into a 20 Vpp sine wave and supplied to the excitation electrode. The resulting current at the sensing electrode signal was converted to voltage and was amplified ~100×. A peak detector circuit was used to output a stable DC signal which was fed into the Arduino NANO analog pin and displayed on an Apple Macbook Pro laptop in real time. The data post-processing included offset correcting the voltages corresponding to DI water (0 μM concentration) to 0 V, acting as the one-step calibration of the sensor, and was followed by signal-to-noise (S/N) measurements. The lower limit of detection (LOD) was calculated by linear fitting the data and calculating the concentration at a signal-to-noise ratio (SNR) = 3. After a month of benchtop storage under normal laboratory conditions, the devices were re-tested for deterioration in performance.

**Figure 2:**
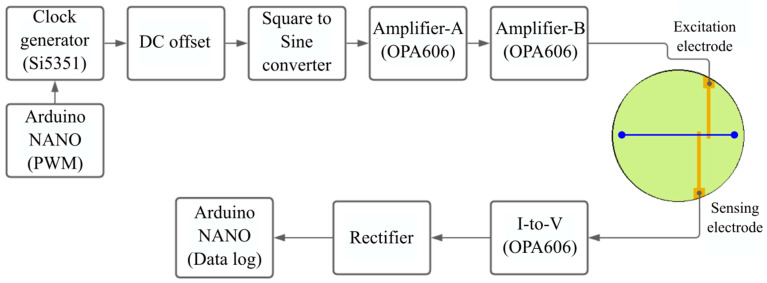
Schematic diagram of the C^4^D design hardware flowchart. A single-stage amplification is, in theory, sufficient for amplifying the excitation signal. However, we observed noise beyond a certain point of amplification in Amplifier-A, therefore requiring two amplification stages (A and B) to achieve the necessary excitation signal amplitude. This may be unique to our circuitry. The circuit is self-sufficient and is powered by the NANO board.

All operational amplifiers were OPA606KPs (Texas Instruments, Dallas, TX, USA) and biased using simple dc-dc converters (Garosa, Amazon store, Seattle, WA, USA). All wires, resistors, capacitors, and diodes were purchased from Mouser electronics (Mansfield, TX, USA). The excitation signal and the final output signal were monitored in parallel on the digital phosphor oscilloscope (DPO3012, Tektronix, Beaverton, OR, USA) to detect circuit anomalies. The flowchart of the hardware components is shown in Figure 2, and the entire schematic is shown in Supplementary Materials Figure S2.

### 3.3. SIP Subpayload

#### 3.3.1. SIP Component Selection Rationale

The initial impact tests in our previous work used piezo actuators (PN: 810-10, PI, Auburn, MA, USA) [18]. They were cylindrical in shape and had a shoulder–actuating disk area ratio of 4:5 ((9*π*-4*π*) mm^2^/4*π* mm^2^) meaning only 20% of the solenoid could be supported by the shoulder, as opposed to the shoulder–piston rod base area percentage of 94.6% ((132-2.25*π*) mm^2^/132 mm^2^) of the solenoid actuators used in this work. Due to the small shoulder on the PI solenoids, they had to be press-fitted into the actuator slots in the first design iteration. The impact tests showed a displacement of the actuators during the impact event and fractured the glass microfluidic chip [18]. Though the actuators used in this work are slightly larger (6 mm diameter cylinder versus 12 mm wide cuboid), they do not need to be press-fitted inside the canister. Adding to this advantage, the solenoid construction is simplistic with just a coil wrapped around a plunger, has very few failure modes, and therefore may have a better impact resistance. To prepare the test article for impact tests, electronic boards in the hollow region of the canister could be encapsulated with a polyurethane potting compound (Cytec, Part name: CONATHANE (EN-1556), Ellsworth, Germantown, WI, USA) to mitigate damage during launch, impact, and other physical stresses, as demonstrated by Cato et al. [18].

#### 3.3.2. SIP Microfluidic Path Design

**Figure 3:**
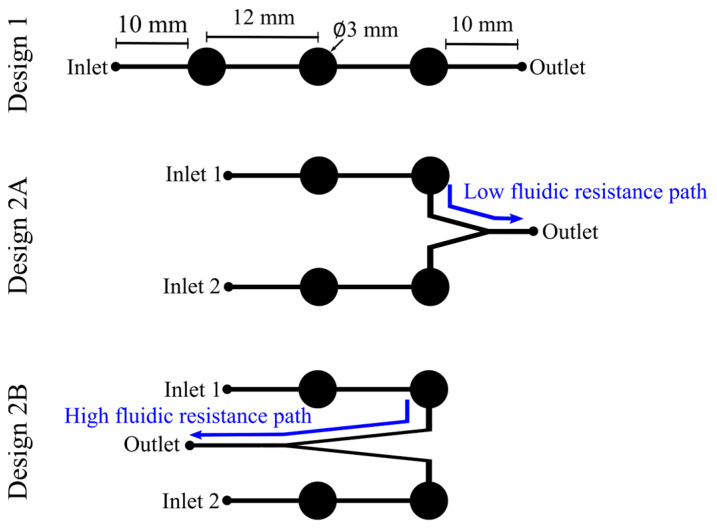
Actuation channel design comparison. The lines represent the microfluidic channels, and the circles represent the actuation pads. Design 1 was the layout used for the single-channel actuation mode. For the two-channel design, two microfluidic layouts were tested—low-fluidic-resistance path design (2A) and high-fluidic-resistance path design (2B).

Two types of microfluidic path designs were tested in this work—single-channel and two-channel. The single channel was a trivalve design (design 1) and had the three valves placed in a series (Figure 3), the two-channel design had two bivalve designs, each with different outlet port placements, named 2A and 2B (Figure 3). Pumping protocols were examined for all designs. For design 1, the solenoids were actuated from left to right to pump the fluid from the inlet to the outlet. For designs 2A and 2B, the pumping protocol was in two stages; in the first stage, the two solenoids on the active channel (channel 1) were actuated in a sequence, and the two solenoids on the inactive channel (channel 2) were activated to close the channel. In the second stage, the protocol was reversed to make channel 2 active and channel 1 inactive. Exploring two-channel geometry was necessary to enable the one-step calibration of the C^4^D sensor—inlet 1 for the DI water sample from Earth acting as the blank and inlet 2 for the sample internalized from Europa.

#### 3.3.3. SIP Microchannel Geometry

Establishing the microchannel geometry and the actuation pad thickness was the topmost priority to ensure successful fluid actuation with the solenoid-based actuators chosen for this work. First, three trivalve designs with varying sizes of actuation pads were fabricated using the steps mentioned above. The effective length of the channel was kept constant at 4.5 cm and was constrained by the IceShIP canister dimensions. Three actuation pad sizes were tested—2 mm, 3 mm, and 4 mm—guided by the solenoid plunger base with a diameter of 3 mm. The distance between the circular actuation pads on the microchannel was determined based on the distance between the solenoid plungers when placed directly next to each other.

#### 3.3.4. SIP Actuation Pad Layer Fabrication

This work used a pumping mechanism with direct mechanical force on the actuation pad to move the liquid. The structure was completely made of polymer, and the valve type was normally open and required a push from the solenoid plunger to close the valve; the thickness of the actuation layer was therefore critical. Four layers with varying thicknesses were fabricated using the steps mentioned in Govinda Raj et al. [32]. The thicknesses ranged from ~1 mm to ~4 mm by controlling the amount of polymer base + curing agent mixture poured into the acrylic mold. Each layer was tested for a total of 10 cycles.

#### 3.3.5. SIP Solenoid Actuation Software and Hardware Setup and Operation

An Arduino NANO (ATmega328) was custom-programmed using the Arduino platform installed on an Apple Macbook Pro laptop. The code was used to generate pulse-width-modulated (PWM) signals of 300 ms on time on four analog pins in a loop function, facilitating a simple “on–wait–off–wait” sequence. These digital command signals were converted into analog voltages to actuate four mini push–pull solenoids (PN: 2776, Adafruit, New York, NY, USA). The COTS Arduino NANO board came with a crystal resonator and enabled an extremely accurate time delay between the on/off times, ±20 ppm at 16 MHz (2.5 ps), and did not have observable effects on the solenoid response time [36]. The solenoids were positioned directly above the actuation pads with less than 2 mm vertical clearance between the plunger in the off state and the PDMS actuation pads underneath. Pressing down on the circular actuation pads (Figure 3) in a sequential fashion created a peristaltic pump, effectively moving the fluid. The circuit schematic for the solenoid circuitry is shown in the Supplementary Materials Figure S3. Using a micropipette, a 50 µL dyed water droplet was placed at the inlet(s); a smartwatch timer was turned on when the software code was initiated and turned off upon complete fluidic transfer without residue in the channel.

### 3.4. MicroICE and SIP Hardware Machining Specifications

The redesigned canister housing for all components was machined on a knee mill (Trak, Southwestern Industries, Inglewood, CA, USA) and a lathe (Grizzly, Bellingham, WA, USA) using 2.5″ diameter 7075 aluminum bars.

**Figure 4:**
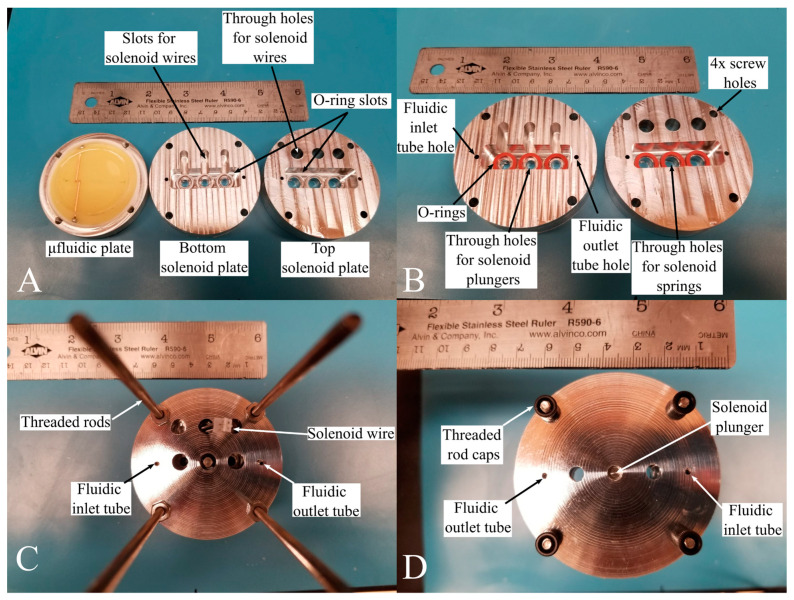
Solenoid placement inside the canister. (**A**) The solenoid holding plates are directly above the microfluidic plate. The microfluidic plate houses a press-fitted PTFE cup holding the C^4^D disk. (**B**) Top and bottom plates of the solenoid holder designed with slots for wires and o-rings. (**C**) Top view of the solenoid installed in the holder. (**D**) Bottom view of the solenoid installed in the holder.

The remodeled canister had the same diameter as the older versions [18] but had added height to host the new SIP and MicroICE hardware components. The MicroICE device disk was press-fitted inside a custom-made polytetrafluoroethylene (PTFE) cup, with two aluminum plates housing the SIP solenoids right above and the supporting electronics segment at the very top. The two solenoid housing plates were designed to hold the commercial off-the-shelf (COTS) solenoids perfectly aligned above the microchannel actuation pads (Figure 4) with no room for misalignment—the bottom plate had slots for the wires and o-rings, and the top plate had holes for the spring-plunger arrangement during the solenoid off state and holes for the solenoid wires so they could be connected to the electronic hardware segment above. The canister computer-aided designs (CADs) are shown in Figure S4 in the Supplementary Materials section.

The body components were screwed together by black-oxide-treated alloy steel cap-head bolts installed through mounting holes and threaded into the base plate. All hardware machining parts were purchased from McMaster-Carr (Elmhurst, IL, USA).

## 4. Results and Discussion

### 4.1. MicroICE Subpayload Results

#### 4.1.1. MicroICE Device Characterization

The average MicroICE electrode width was 710 ± 40 μm, and the average electrode separation distance was 1360 ± 60 μm. The PDMS insulating layer thickness was 100 ± 10 μm. ANOVA analyses comparing the voltage readings for all four salts, for all trials, on the three identically fabricated MicroICE devices showed no statistically significant differences in the performance (confidence interval (CI) = 95%, α = 0.05). A voltage offset was required when the setup was used after benchtop storage for a month, but after this calibration step, Student T-tests showed no statistically significant differences in the device performances despite the time in storage (CI = 95%, α = 0.05) [32].

#### 4.1.2. MicroICE Device Miniaturization Characterization

**Figure 5:**
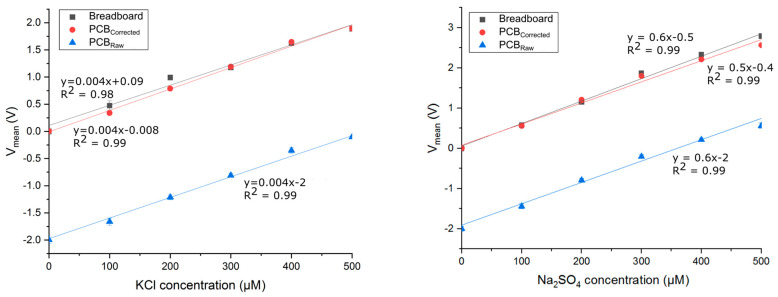
Comparing voltage responses of breadboard and PCB hardware versions with KCl and Na_2_SO_4_ salts. The hardware circuits were tested with the same C^4^D device. The PCB hardware needed an offset correction of +2V after which the voltage response was similar to the breadboard version and showed no loss of performance as determined by Student T-tests. “Breadboard” curves are the readings from the breadboard setup; “PCB_Raw_” are the readings from the PCB setup before offset correction; “PCB_Corrected_” are the offset-corrected readings to match the breadboard voltage offset.

The breadboard and the PCB hardware were tested on Device 2 for KCl and Na_2_SO_4_ salts (Figure 5). Due to variations in internal capacitances and wire resistances, it was observed that the PCB hardware needed a positive offset correction (+2 V) as opposed to the negative offset correction in the breadboard setup, but after this correction, the Student T-test showed no statistically significant differences.

#### 4.1.3. MicroICE LOD Measurement Results

The linear range (LR) for all four salts was established first. The LR was found to be 50 µM–1000 µM for NaCl salt and was 50 µM–500 µM for the other three salts. Therefore, for LOD experiments, we freshly prepared samples spaced 50 µM apart, within a 50 µM–500 µM range for all four salts to maintain uniformity. The LOD values for all four salts across three trials on three devices are shown in Table 1.

#### 4.1.4. “Real-World” Sample Testing on the MicroICE

**Figure 6:**
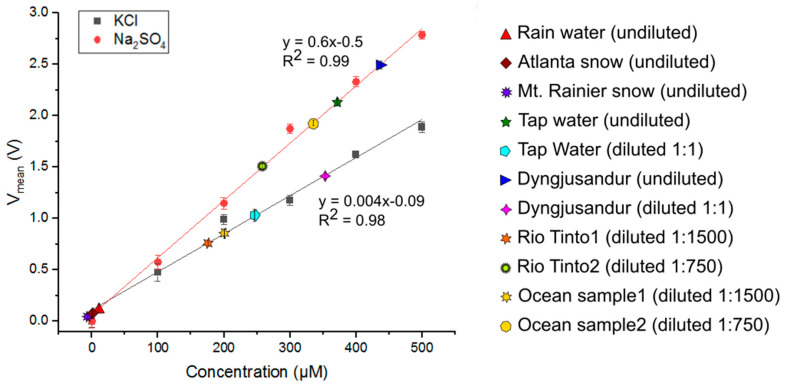
Seven real-world samples tested on Device 2—tap water from the lab faucet, rainwater, Mt. Rainier snow, Atlanta snow, Rio Tinto water, sediment samples from Dyngjusandur, and ocean water samples. For device validation, rainwater, Atlanta snowmelt, and Mt. Rainier snowmelt samples were used without dilution, tap water and Dyngjusandur samples were used without dilution first and then diluted 1:1, and Rio Tinto and ocean samples were diluted 1:750 and 1:1500. All real-world sample voltages were plotted against KCl and Na_2_SO_4_ curves to estimate the total dissolved solid (TDS) content.

Seven “real-world” samples were tested on the MicroICE Device 2 and plotted against KCl and Na_2_SO_4_ calibration curves to estimate their total dissolved solid (TDS) content (Figure 6). Their dilution ratios and corresponding concentrations are tabulated in Table 2. Rainwater, snowmelt, and Mt. Rainier are examples of pure water sources; they have very low TDS concentrations and are essentially as pure as DI water. Tap water is processed water and tends to have a low TDS content [37]. Dyngjusandur is a lava field in Iceland and is considered geologically analogous to Mars [38]. The ocean water sample is an example of a high-salinity sample and contains a complex mixture of high amounts of salts [39], and it could be analogous to the Europan ocean [40]. Rio Tinto is an example of both high salinity and extreme acidity. It contains high amounts of iron, magnesium, copper, zinc, and salts [41] and a pH of <2, making it a suitable analog sample for Europan surface conditions [42].

Rio Tinto has an electrical conductivity of ~40 mS/cm (25,600 ppm, from Equation (1)) [41]. The 40 mS/cm converts to 343 mM for KCl and 180 mM for Na_2_SO_4_ (Equation (2)). The ocean sample has an electrical conductivity of ~48 mS/cm (30,400 ppm) [43]. The 48 mS/cm converts to 408 mM for KCl and 214 mM for Na_2_SO_4_ [44]. From the corresponding concentration values, the TDS contents of Rio Tinto and the ocean sample are tabulated in Table 2. The ranges measured in this work are well within the range of conductivity values reported by other groups that used commercial probes and hence validate the device performance.

### 4.2. SIP Subpayload Results

#### 4.2.1. SIP Microfluidic Path Design Characterization

The pumping protocols examined in this work are shown in Figure S1 in the Supplementary Materials along with the fluidic behavior inside the channel. The blue lines are the pulse-width-modulated signals with an on time of 300 s and indicate the solenoid status—flatline shows an inactive solenoid, and a raised pulse indicates an active solenoid. A completely black channel represents a channel filled with fluid, and a gray channel represents an empty channel. Gradient gray channels show partially filled portions during the actuation sequence. The “t_0_” is the initial state of the solenoids.

For design 1, the solenoids were actuated in a sequence from the left to the right to pump the fluid from the inlet to the outlet. Design 2A had a low-fluidic-resistance path which yielded fast flow rates as anticipated, but due to the low-fluidic-resistance path, backflow from the outlet contaminated the sample in inlet 2 even before channel 2 was activated and despite the closed valves. Design 2B was later adopted to mitigate this contamination issue [23]. The contamination control effort in this work is discussed in more detail in Section 4.2.4.

#### 4.2.2. SIP Actuation Pad Geometry Characterization

**Figure 7:**
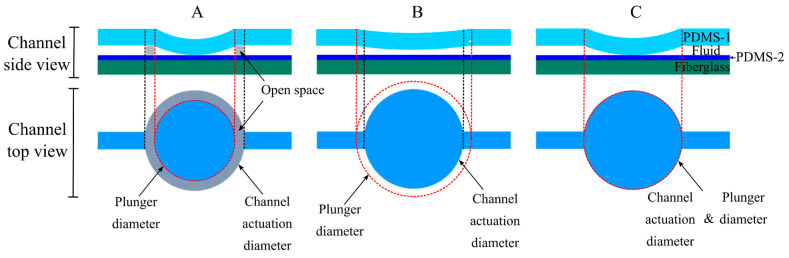
Actuation pad dimensions. The solenoid plunger diameter dictated the actuation pad geometry. Three designs were tested to determine the geometry allowing the best channel closure when not in use. Design (**A**) provided a dead space and permitted fluidic flow even when closed. Design (**B**) did not allow the closure of the valve. Design (**C**) was chosen due to its superior valve closure capabilities.

The three trivalve geometries tested in this work are shown in Figure 7. The goal was to empirically determine the actuation pad geometry that provided complete channel closure when pressed down by the actuators. The actuation pad with a 4mm diameter (Figure 7A) provided a circular dead space, undesirably permitting fluidic flow even when closed. The 2 mm pad (Figure 7B) did not allow full channel closure.

The 3 mm diameter actuation pad (Figure 7C) was chosen given its complete closure capabilities consistently in all trials. Figure 7 gives a pictorial representation of this capability to avoid leaks within the channel. The separation distance between the actuation circles was determined based on the distance between the solenoid plungers when placed right next to each other.

#### 4.2.3. SIP Actuation Pad Layer Thickness Characterization

The single-channel trivalve design was used to check for valve closure capacity with varying actuation layer thickness. The results are shown in Table 3. For all cases in which fluidic flow was observed, the flow rate remained constant. It was found that slabs with a 2–3 mm thickness were the most structurally robust and gave the most consistent pumping routines. All further devices were fabricated maintaining this thickness range.

#### 4.2.4. SIP Microchannel Contamination Control

**Figure 8:**
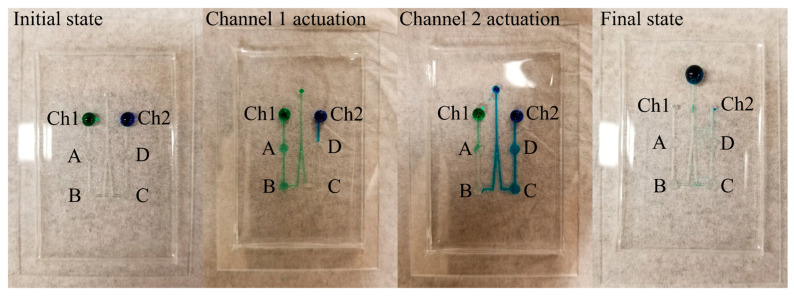
Food coloring used as dye to visibly examine fluidic flow and back contamination. The **initial state** has green and blue colors dropped at the two channel inlets. During **channel 1 actuation**, it was observed that a small portion of the blue dye had flowed into the channel but not beyond the first actuation point (D). Also, a slight backflow of green dye occurred but not beyond the second actuation point (C). Next, during **channel 2 actuation**, a similar backflow of blue dye was observed, but it did not go beyond the second actuation point (B). Green dye was observed to occupy the first actuation point on channel 1 (A), but it stopped there, and no cross-contamination was observed. In channels 1 and 2, the backflow stopped at the valves even when the cycles were repeated three times more than the protocol requirement.

Due to the extreme sensitivity of the C^4^D setup, avoiding backflow and the cross-contamination of samples from two different inputs was necessary. Design 2B was therefore employed—it had the same solenoid actuation pads, but the outlet channel was lengthened for a high-fluidic-resistance path to avoid backflow and subsequent cross-contamination [23]. In design 2B (Figure 8), during channel 1 actuation, the contamination initially reached the second microvalve (microvalve C) in the inactive channel (channel 2), and simultaneously, the sample from inlet 2 was observed to move towards microvalve D, but neither moved beyond microvalves C and D even after 3x the number of pumping cycles necessary to move the sample from inlet 1 to the outlet.

Similarly, during channel 2 actuation, the channel 1 content stagnated near microvalve A, and the contamination from channel 2 reached the second microvalve (microvalve B) in the inactive channel (channel 1), but neither sample moved beyond microvalves B and A even after 3× the number of pumping cycles necessary to move the sample from inlet 1 to the outlet. In the final state, the entire volume of both samples had reached the outlet. The positive results from this test prepared the microchannel design for integration with the MicroICE setup.

### 4.3. MicroICE and SIP Integration

Once the microfluidic channel design was optimized, design 1 and design 2B were integrated with the MicroICE sensor setup. The C^4^D electrodes work in contactless mode and therefore need excitation amplitudes in the 10s of volts range. These voltages invariably create a magnetic field around the electrode region. It was observed that a human hand within 10 cm of the electrode field altered the voltage readings. Solenoids, on the other hand, convert electrical energy to mechanical motion using magnetically movable plungers. A magnetically active component within the C^4^D electrode field was expected to interfere with the sensor readings.

**Figure 9:**
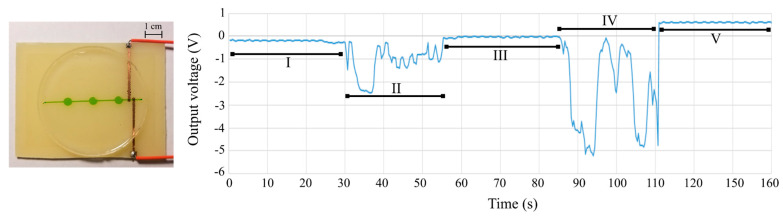
Integrated automated actuation and MicroICE data on the single-channel device. (**Left**) Single-channel, trivalve linear channel aligned with C^4^D electrodes. The channel is filled with dye for better visualization. (**Right**) Real-time C^4^D data. The regions in the plot are delineated in Table 4.

As a workaround to this, for design 1, the solenoid code was written to first move the sample from the inlet toward the outlet by working in an “on–wait–off–wait” sequence for 25 sec, and then, the sample was “stagnated” above the electrodes for 30 sec during which the solenoids were turned off for C^4^D data recording. After this 30 sec pause, the solenoids were turned back on to move the sample completely to the outlet while also pumping in the new sample that was manually placed at the inlet. The C^4^D response for design 1 is shown in Figure 9, and the solenoid statuses are tabulated in Table 4.

**Figure 10:**
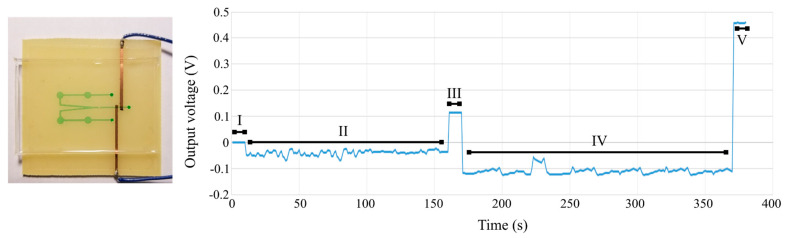
Integrated automated actuation and MicroICE data on the two-channel device. (**Left**) Two-channel, bivalve outlet channel aligned with C^4^D electrodes. The channel is filled with dye for better visualization. (**Right**) Real-time C^4^D data. The regions in the plot are delineated in Table 5.

For design 2B, the on and off times had to be different given the change in the channel geometry and the number of solenoids involved in each pumping cycle. The solenoid code was written to first move the sample from the first inlet towards the outlet by working in an “on–wait–off–wait” sequence for 150 s spanning 60 cycles to bring the sample above the electrodes, and then, the solenoids entered the idle mode for 10 s during which the solenoids were turned off. After this 10 s pause, the solenoids in the second channel were turned on to move the old sample from the electrode field while also pumping in the sample from the second inlet towards the outlet for 150 more seconds. The C^4^D response for design 2 is shown in Figure 10, and the solenoid statuses are tabulated in Table 5.

A 300 s solenoid delay in the code produced the most consistent pumping cycles. The C^4^D code for both pumping routines was the same and was written to collect the voltage readings uninterruptedly throughout the cycles. During the solenoid on time, the C^4^D readings were subject to significant interference and therefore had no electrochemical significance, but during the “solenoid idle” mode, the voltage readings were as expected and matched the calibration curves.

### 4.4. IceShIP Canister Redesign Specifications

The IceShIP canister required minor design modifications to host the added system components.

**Figure 11:**
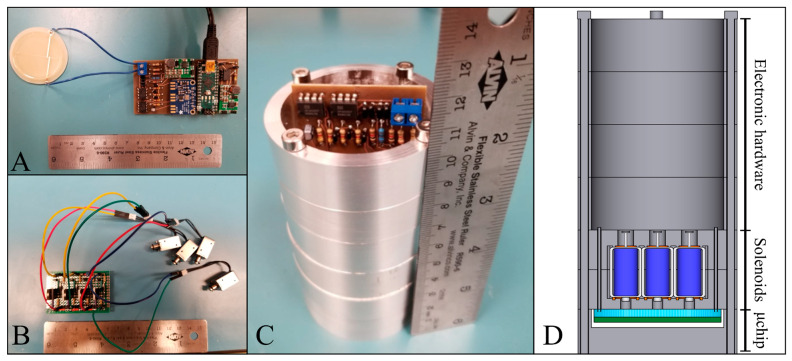
(**A**) C^4^D PCB connected to the electrode disc. (**B**) Actuator perfboard connected to the solenoids. (**C**) Circuit boards placed inside the canister. (**D**) Corresponding CAD design showing the placement of components inside the canister.

Figure 11 shows the printed circuit boards of the MicroICE and the SIP hardware fitting inside the remodeled canister. The COTS solenoids were 12.6 g each, and with the PCB, the total mass was 76 g. The canister plates housing the solenoid assembly added a mass of 226 g. The MicroICE setup along with the PCB added 46 g. In all, the MicroICE setup with the SIP setup added 348 g to the IceShIP payload, consuming 2.5 W.

The total cost of all COTS parts was ~USD 80, and the custom-made parts did not require complex fabrication/machining procedures. The all-polymer microchannel design had two major advantages—it eliminated the need for glass wafers and was easy to fabricate—making it well suited for high g load penetrator designs for planetary exploration.

The MicroICE prototype was demonstrated at TRL 3 and tested under controlled conditions in a lab environment; more work is necessary under more representative conditions with simulated impact scenarios for TRL elevation. By extension of this point, the field samples were prepared for analysis in a lab; analyzing true field samples will require additional device capabilities including but not limited to filtration to remove suspended particles, pre-dilution before introducing highly concentrated samples to the system, and sample temperature correction. The limitations discussed here highlight areas for future development, without detracting from the primary contributions of this work, namely demonstrating the feasibility of miniaturized instrumentation for high-acceleration penetrator missions. By addressing these challenges in microfluidic technology and electronic hardware integration, this study lays a solid foundation for the future development of microfluidics-based exploratory platforms.

## 5. Conclusions

Planetary missions to worlds with exciting astrobiological significance need access to subsurface samples for habitability analyses and can require complex, high-powered soft-lander platforms that are large, heavy, and fragile. Penetrator missions equipped with analytical instruments fitting within low-volume, -mass, and -power-consumption envelopes have a great potential for enabling robust, topographically distributed, low-cost missions. IceShIP is a first-of-its-kind science payload platform for state-of-the-art analytical instrumentation employing LIF for the detection of low-concentration organics and C^4^D for the detection of inorganic species on a programmable microfluidic platform enabled by SIP. The design is miniaturized and consumes low power, making it ideal for applications with small payload footprint requirements to meet the arduous demands of planetary science missions.

We report the performance of a miniaturized MicroICE device and its integrated operation with a novel automated two-channel fluid routing mechanism on a microfluidic platform. MicroICE employs the principle of contactless conductivity detection for quantifying low-concentration inorganics and is capable of reaching tens of micromolar LODs for four Europa-relevant salts, exceeding the NASA-recommended LOD by four orders of magnitude. Seven “real-world” samples were tested to validate the device’s performance. Automated sample routing was achieved using the custom programming of mini COTS solenoids actuating a programmable microfluidic architecture called SIP. Custom-machined plates for the COTS solenoid holders were made to integrate the design with IceShIP, adding a mass of only 348 g to the IceShIP canister. The setup is at TRL 3 and demonstrates a low-mass, small-volume, and low-power instrument design geared towards high-acceleration space flight missions.

In the future, the integration of the detection technique with a separation technique like microchip capillary electrophoresis is imperative for the identification of ionic species. This must be followed by hardware miniaturization for integration into the IceShIP canister. The technology readiness level of this model will reach a true TRL 4 from the current TRL 3 with impact tests confirming the physical and functional survivability of the components.

Multiple IceShIP payloads could be used in a Discovery-class orbiter mission and be ejected to carry out analyses on samples with significant geographical spacing without roving capabilities. This prototype demonstration proves the merit of further work to elevate the TRL of this MicroICE + SIP instrument and IMPOA subpayloads as a whole via further mission-targeted design, build, and testing.

## Figures and Tables

**Table 1:** Lower limit of detection of all three devices for four Europa-relevant salt analytes [32].

LOD (µM)	NaCl	MgSO_4_	Na_2_SO_4_	KCl
**Device 1**	80 ± 30	20 ± 10	40 ± 20	100 ± 20
**Device 2**	100 ± 50	30 ± 10	20 ± 10	60 ± 10
**Device 3**	70 ± 30	60 ± 30	40 ± 10	100 ± 10

**Table 2:** Seven “real-world” samples were tested on the MicroICE Device 2 and plotted against KCl and Na_2_SO_4_ calibration curves to estimate their total dissolved solid (TDS) content.

Sample	Dilution Ratio	MicroICE Calibration Curve Data		Literature Values
		Conc. Values	Conc. Range Estimate	
**Rainwater**	-	10 μM	10 μM	-
**Atlanta snow**	-	0	0	-
**Mt. Rainier snowmelt**	-	0	0	-
**Tap water**	-	375 μM	375–500 μM	-
**Diluted tap water**	1:1	250 μM		
**Dyngjusandur sediment sample water**	-	425 μM	425–700 μM	-
**Diluted Dyngjusandur sediment sample water**	1:1	350 μM		
**Diluted Rio Tinto water 1**	1:1500	175 μM	206–263 mM	180–343 mM
**Diluted Rio Tinto water 2**	1:750	275 μM		
**Diluted ocean sample 1**	1:1500	200 μM	263–300 mM	214–408 mM
**Diluted ocean sample 2**	1:750	350 μM		

**Table 3:** Microchannel slab thickness optimization.

Thickness (mm)	Flow Rate Consistent?	Observation
0.96	Yes	Fluidic flow occurred, but the slab ruptured and caused a leak in the microchannel during the fifth cycle.
2.11	Yes	Fluidic flow occurred. No rupture observed. Structural integrity preserved even after 10 cycles.
2.94	Yes	Fluidic flow occurred. No rupture observed. Structural integrity preserved even after 10 cycles.
4.07	-NA-	No fluidic flow. Slab too thick to deflect under solenoid push.

**Table 4:** Design 1 (single-channel, trivalve) solenoid actuation sequence.

Region	Duration (s)	Channel Content	Solenoid Status
I	-	Dry	Off
II	25	Dry (DI inlet)	On
III	30	DI	Off
IV	25	DI flush/300 µM inlet	On
V	30	300 µM NaCl	Off

**Table 5:** Design 2B (two-channel, bivalve) solenoid actuation sequence.

Region	Duration (s)	Channel Content	Solenoid Status
I	-	Dry	Off
II	150	Dry (DI inlet)	On
III	10	DI	Off
IV	200	DI flush/300 µM inlet	On
V	10	300 µM NaCl	Off

## Data Availability

Data are contained within the article and supplementary materials.

## Supplementary Materials

The following supporting information can be downloaded at: https://www.mdpi.com/article/10.3390/s24237704/s1.

## References

[1] Buratti B.J. (1995). Photometry and surface structure of the icy Galilean satellites. J. Geophys. Res. Planets.

[2] Carr M.H., Belton M.J.S., Bender K., Breneman H., Greeley R., Head J.W., Klaasen K.P., McEwen A.S., Moore J.M., Murchie S. (1995). The Galileo Imaging Team plan for observing the satellites of Jupiter. J. Geophys. Res. Planets.

[3] Hendrix A.R., Hurford T.A., Barge L.M., Bland M.T., Bowman J.S., Brinckerhoff W., Buratti B.J., Cable M.L., Castillo-Rogez J., Collins G.C. (2018). The NASA Roadmap to Ocean Worlds. Astrobiology.

[4] Hand K.P. (2017). Report of the Europa Lander Science Definition Team.

[5] McKay C.P., Khare B.N., Amin R., Klasson M., Kral T.A. (2012). Possible sources for methane and C2–C5 organics in the plume of Enceladus. Planet. Space Sci..

[6] Pizzarello S. (2006). The Chemistry of Life’s Origin: A Carbonaceous Meteorite Perspective. Acc. Chem. Res..

[7] Duca Z.A., Speller N.C., Cantrell T., Stockton A.M. (2020). A modular, easy-to-use microcapillary electrophoresis system with laser-induced fluorescence for quantitative compositional analysis of trace organic molecules. Rev. Sci. Instrum..

[8] Neveu M., Hays L.E., Voytek M.A., New M.H., Schulte M.D. (2018). The Ladder of Life Detection. Astrobiology.

[9] Nordheim T.A., Hand K.P., Paranicas C. (2018). Preservation of potential biosignatures in the shallow subsurface of Europa. Nat. Astron..

[10] Brown M.E., Hand K.P. (2013). Salts and Radiation Products on the Surface of Europa. Astron. J..

[11] Trumbo S.K., Brown M.E., Hand K.P. (2019). Sodium chloride on the surface of Europa. Sci. Adv..

[12] Lane A.L., Nelson R.M., Matson D.L. (1981). Evidence for sulphur implantation in Europa’s UV absorption band. Nature.

[13] Carlson R.W., Anderson M.S., Mehlman R., Johnson R.E. (2005). Distribution of hydrate on Europa: Further evidence for sulfuric acid hydrate. Icarus.

[14] Kargel J.S. (1998). PLANETARY SCIENCE: The Salt of Europa. Science.

[15] McCord T.B., Teeter G., Hansen G.B., Sieger M.T., Orlando T.M. (2002). Brines exposed to Europa surface conditions. J. Geophys. Res. Planets.

[16] Pappalardo R.T., McKinnon W.B., Khurana K. (2009). Europa.

[17] Raj C.G., Cato M., Speller N.C., Duca Z., Putman P., Epperson J., Foreman S., Kim J., Stockton A. (2022). Icy Moon Penetrator Organic Analyzer Post-Impact Component Analysis. Front. Astron. Space Sci..

[18] Cato M., Raj C.G., Speller N., Duca Z., Stockton A., Kim J., Putman P., Epperson J. Icy Moon Penetrator Organic Analyzer (IMPOA) Impact Test Results. Proceedings of the 2022 IEEE Aerospace Conference (AERO).

[19] Gowen R., Smith A., Fortes A., Barber S., Brown P., Church P., Collinson G., Coates A., Crawford I., Dehant V. (2011). Penetrators for in situ subsurface investigations of Europa. Adv. Space Res..

[20] Hopf T., Kumar S., Karl W.J., Pike W.T. (2010). Shock protection of penetrator-based instrumentation via a sublimation approach. Adv. Space Res..

[21] Jensen E.C., Stockton A.M., Chiesl T.N., Kim J., Bera A., Mathies R.A. (2013). Digitally programmable microfluidic automaton for multiscale combinatorial mixing and sample processing. Lab A Chip.

[22] Kim J., Stockton A.M., Jensen E.C., Mathies R.A. (2016). Pneumatically actuated microvalve circuits for programmable automation of chemical and biochemical analysis. Lab A Chip.

[23] Stockton A.M., Mora M.F., Cable M.L., Willis P.A. (2013). Design rules and operational optimization for rapid, contamination-free microfluidic transfer using monolithic membrane valves. Sens. Actuators B Chem..

[24] Purwidyantri A., Ipatov A., Domingues T., Borme J., Martins M., Alpuim P., Prado M. (2022). Programmable graphene-based microfluidic sensor for DNA detection. Sens. Actuators B Chem..

[25] Lin H., Tan J., Zhu J., Lin S., Zhao Y., Yu W., Hojaiji H., Wang B., Yang S., Cheng X. (2020). A programmable epidermal microfluidic valving system for wearable biofluid management and contextual biomarker analysis. Nat. Commun..

[26] Welch D., Christen J.B. A multiparametric biosensor array for on-chip cell culture with feedback controlled microfluidics. Proceedings of the 2011 IEEE International Symposium on Circuits and Systems (ISCAS).

[27] Quang L.D., Bui T.T., Hoang A.B., Van T.P., Jen C.-P., Duc T.C. (2019). Development of a Passive Capacitively Coupled Contactless Conductivity Detection (PC4D) Sensor System for Fluidic Channel Analysis Toward Point-of-Care Applications. IEEE Sens. J..

[28] Paknahad M., Ghafarinia V., Hossein-Babaei F. A microfluidic gas analyzer for selective detection of biomarker gases. Proceedings of the 2012 IEEE Sensors Applications Symposium (SAS).

[29] Gubartallah E.A., Makahleh A., Quirino J.P., Saad B. (2018). Determination of Biogenic Amines in Seawater Using Capillary Electrophoresis with Capacitively Coupled Contactless Conductivity Detection. Molecules.

[30] Kim J., Stockton A.M., Willis P., Lillis R., Amundson R., Beegle L., Butterworth A., Curtis D., Ehrenfreund P., Grunthaner G. The Mars Organic Analyzer: Instrumentation and Methods for Detecting Trace Organic Molecules in our Solar System. Proceedings of the 18th International Conference on Miniaturized Systems for Chemistry and Life Sciences.

[31] Butterworth A.L., Kim J., Stockton A.M., Turin P., Ludlam M., Mathies R.A. Instrument for Capturing and Analyzing Trace Organic Molecules from Plumes for Ocean Worlds Missions. Proceedings of the 3rd International Workshop on Instrumentation for Planetary Missions (2016).

[32] Govinda Raj C., Salyards C., Odeh M., Stockton A.M. Microfluidic Inorganic Conductivity detector for Europa (MicroICE) using capacitive coupling. Presented at the IEEE Aerospace Conference.

[33] Coltro W.K.T., Lima R.S., Segato T.P., Carrilho E., de Jesus D.P., Lago C.L.D., da Silva J.A.F. (2012). Capacitively coupled contactless conductivity detection on microfluidic systems—Ten years of development. Anal. Methods.

[34] Govinda Raj C.G., Salyards C., Odeh M., Speller N., Cato M., Duca Z., Kim J., Putnam P., Epperson J., Stockton A. Enduring High Impact—An Inorganic Detector for the Icy Moon Penetrator Organic Analyzer (IMPOA). Proceedings of the MicroTAS.

[35] Kubáň P., Hauser P.C. (2005). Effects of the cell geometry and operating parameters on the performance of an external contactless conductivity detector for microchip electrophoresis. Lab A Chip.

[36] megaAVR Data Sheet. Microchip, 2018. http://ww1.microchip.com/downloads/en/DeviceDoc/ATmega48A-PA-88A-PA-168A-PA-328-P-DS-DS40002061A.pdf.

[37] Patterson K.Y., Pehrsson P.R., Perry C.R. (2013). The mineral content of tap water in United States households. J. Food Compos. Anal..

[38] Gentry D., Amador E.S., Cable M.L., Cantrell T., Chaudry N., Duca Z.A., Jacobsen M.B., Kirby J., McCaig H.C., Murukesan G. Quantifying Variability and Correlation in Biomarker and Mineralogical Measurements: Lessons from Five Astrobiological Mars Analogue Expeditions in Iceland. Proceedings of the AGU Fall Meeting Abstracts.

[39] Swenson H. (1983). Why Is the Ocean Salty?. https://eric.ed.gov/?id=ED241328.

[40] Johnson P.V., Hodyss R., Vu T.H., Choukroun M. (2019). Insights into Europa’s ocean composition derived from its surface expression. Icarus.

[41] Aguilera A., Manrubia S.C., Gómez F., Rodríguez N., Amils R. (2006). Eukaryotic Community Distribution and Its Relationship to Water Physicochemical Parameters in an Extreme Acidic Environment, Río Tinto (Southwestern Spain). Appl. Environ. Microbiol..

[42] Stockton A.M., Chiesl T.N., Lowenstein T.K., Amashukeli X., Grunthaner F., Mathies R.A. (2009). Capillary Electrophoresis Analysis of Organic Amines and Amino Acids in Saline and Acidic Samples Using the Mars Organic Analyzer. Astrobiology.

[43] Tyler R.H., Boyer T.P., Minami T., Zweng M.M., Reagan J.R. (2017). Electrical conductivity of the global ocean. Earth Planets Space.

[44] Keenan C., Wood J., Kleinfelter D. (1976). General College Chemistry.

