# Supplementary material for: Early Technology Readiness Level (TRL) Development of the Microfluidic Inorganic Conductivity Detector for Europa and the Solenoid-Based Actuator Assembly for Impact Penetrators
Source: Sensors (Basel). 2024 Dec 2;24(23):7704. doi: 10.3390/s24237704 (PMC11644930; doi:10.3390/s24237704)
Supplement: Supplementary file 1 [file sensors-24-07704-s001.zip › sensors-3316763-supplementary.pdf]

Early TRL Development of the Microfluidic Inorganic Conductivity Detector for Europa (MicroICE) and the Solenoid-based actuator assembly for Impact Penetrators (SIP)

SUPPLEMENTARY MATERIAL

- Commercial TDS meters measure the electrical conductivity of a solution and display the result in ppm using the following formula:

$$\boldsymbol{ppm_{640} = 640 \times \sigma}$$

(Eq. S1)

$\boldsymbol{\sigma}$ is the solution conductivity in mS/cm

- Molarity was calculated from ppm for each salt species using the following formula:

$$X\ moles/L = ppm_{640}/(molar\ mass \times 1000)$$

(Eq. S2)[44]

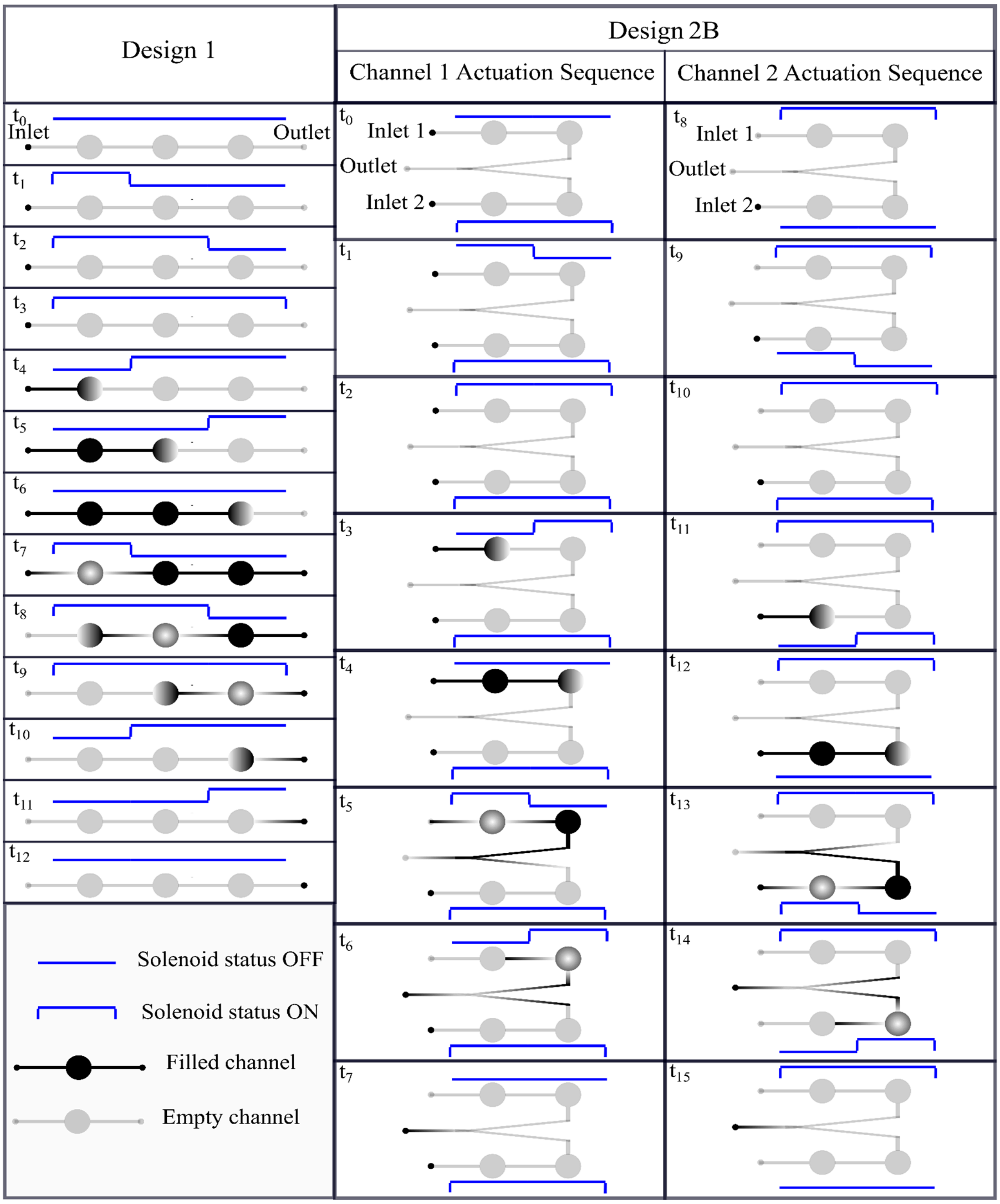

Figure S1: Pumping protocols examined in single-channel (trivalve) and two-channel (bivalve) devices. Blue lines indicate the solenoid status. A black channel represents a channel filled with fluid and a gray channel represents an empty channel. Gradient gray channels show partially filled portions during the actuation sequence. The "t_0" is the initial state of the solenoids. (Left) Design 1 layout with three valves and their corresponding solenoid states mentioned above them. The solenoids are actuated in a left to right sequence and pump the fluid from the inlet to

the outlet. (Right) Design 2B layout with four valves, two on each channel, and their corresponding solenoid states mentioned above and below them. The pumping protocol was in two stages; in the first stage, the two solenoids on the active channel (channel 1) were actuated one by one and the two solenoids on the inactive channel (channel 2) were activated to close the channel. In the second stage, the protocol was reversed to make channel 2 active and channel 1 inactive.

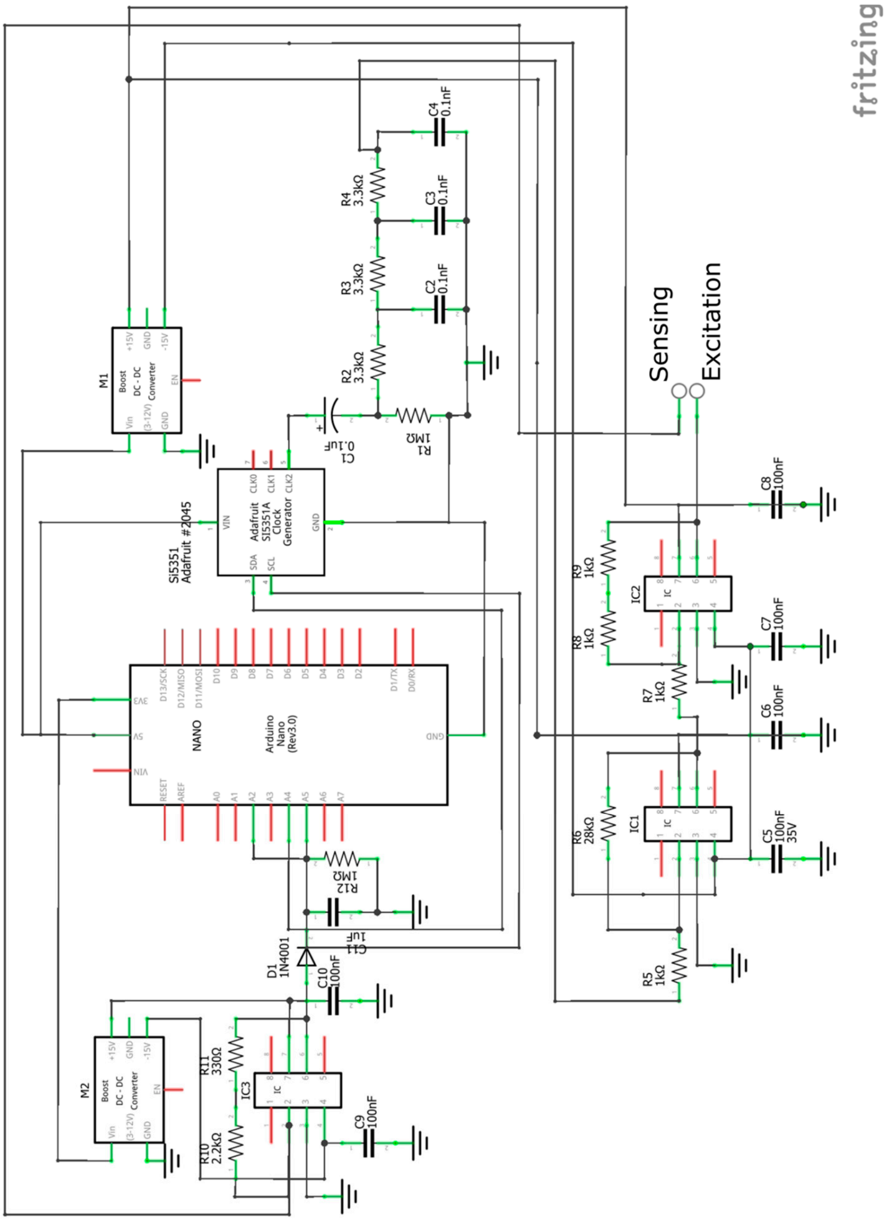

Figure S2: Circuit schematic diagram of the C^4D setup.

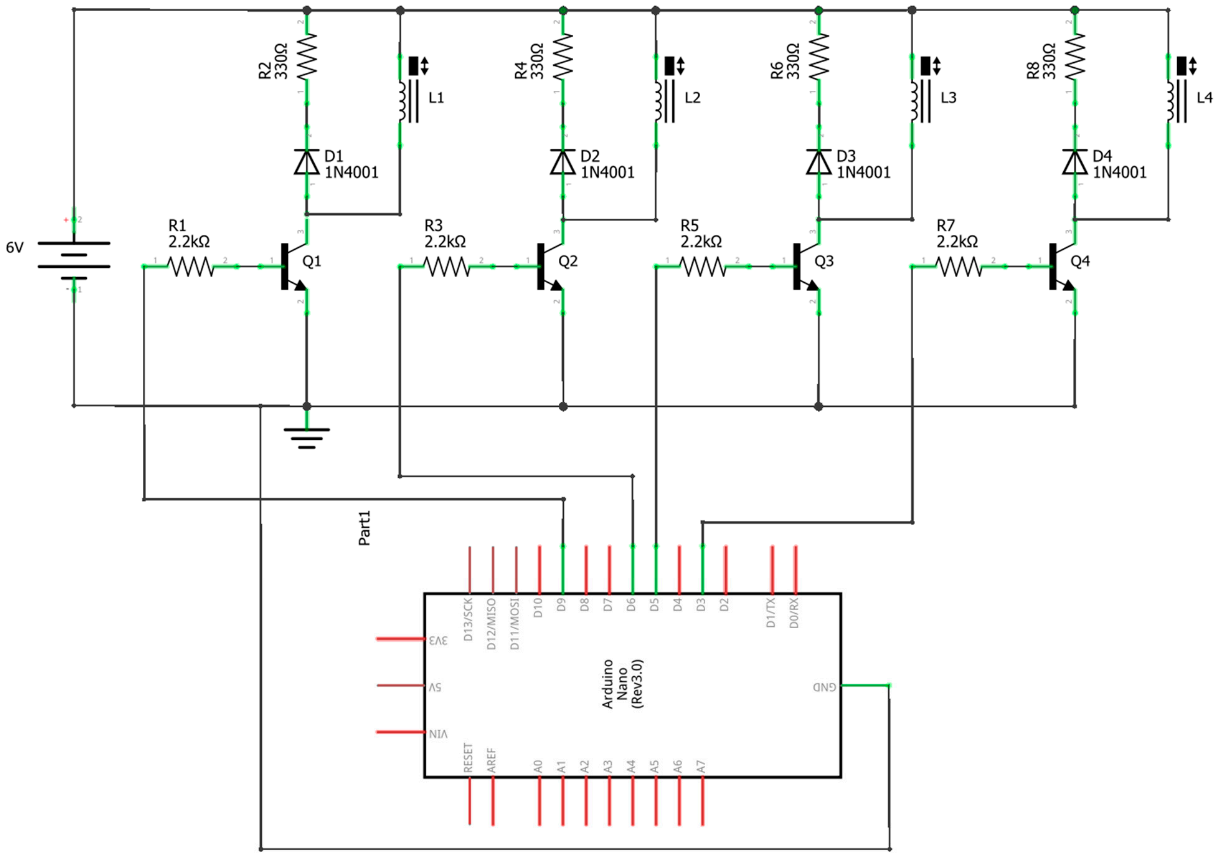

Figure S3: Circuit schematic diagram of the solenoid actuation setup.

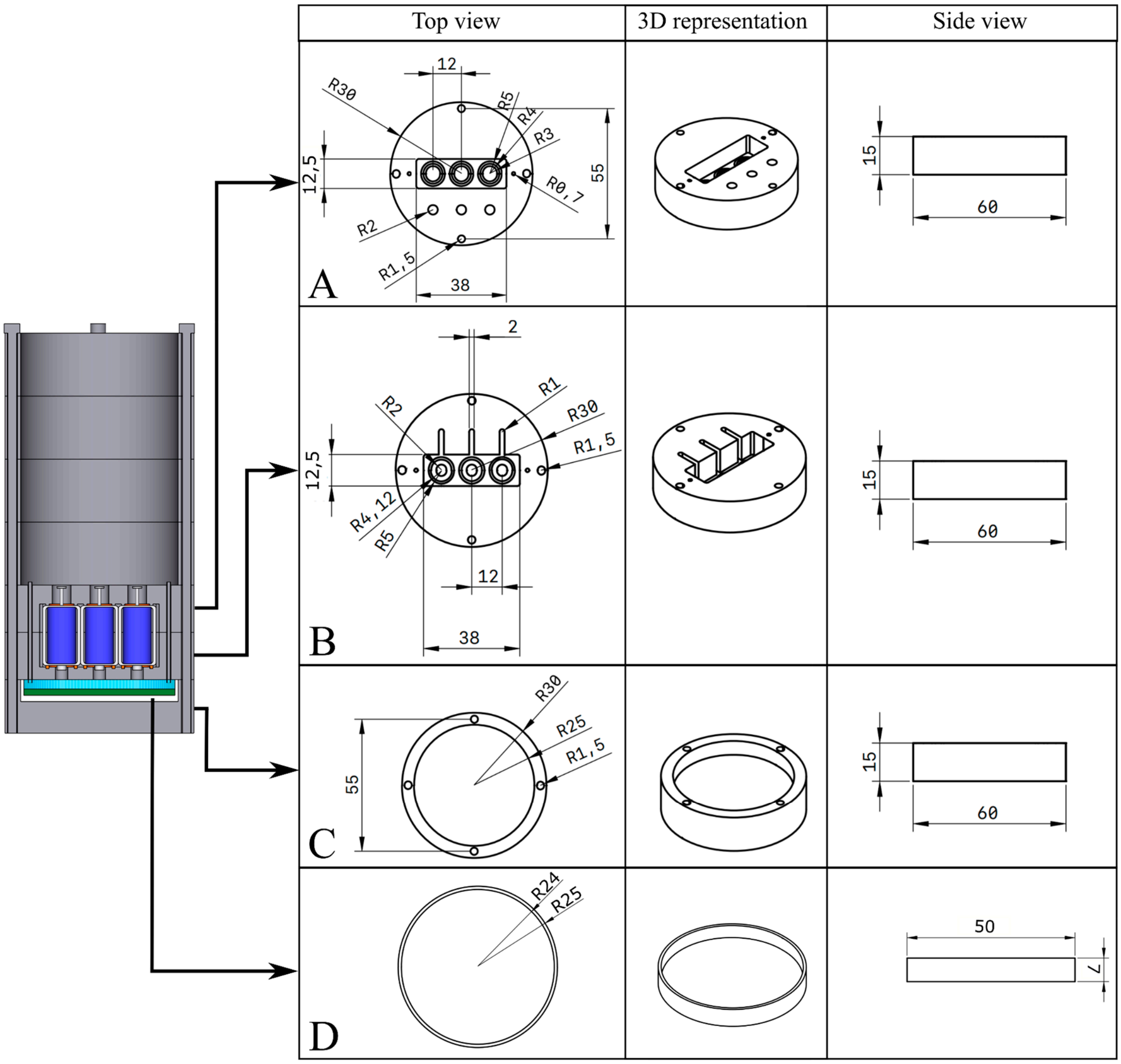

Figure S4: CAD designs with the dimensions annotated in millimeters. (A) Top plate of the solenoid holder. Circular groves are for solenoid o-rings, holes are for the plunger spring in OFF position. Two holes along the side for the tubes connected to the main microfluidic device, and a rectangular slot for half of the solenoid body and electrical collections to connect to the electronic hardware portion of the canister. (B) Bottom plate of the solenoid holder. Has circular grooves for the solenoid o-rings, holes for the solenoid plunger in ON position, holes for the microfluidic device tube connections, and slots for keeping the solenoid wires in place. (C) Bottom plate of the canister. Has a cylindrical grove for holding the PTFE cup. (D) PTFE cup matching the dimensions of the FR-4 disk. All plates A through C have four screw holes. Plate C has tapped holes.
